# Social Determinants of Hallway Bed Use

**DOI:** 10.5811/westjem.2020.4.45976

**Published:** 2020-06-24

**Authors:** David A. Kim, Leon D. Sanchez, David Chiu, Ian P. Brown

**Affiliations:** * Stanford University, Department of Emergency Medicine, Palo Alto, California; † Beth Israel Deaconess Medical Center, Department of Emergency Medicine, Boston, Massachusetts

## Abstract

**Introduction:**

Hallway beds in the emergency department (ED) produce lower patient satisfaction and inferior care. We sought to determine whether socioeconomic factors influence which visits are assigned to hallway beds, independent of clinical characteristics at triage.

**Methods:**

We studied 332,919 visits, across 189,326 patients, to two academic EDs from 2013–2016. We estimated a logistic model of hallway bed assignment, conditioning on payor, demographics, triage acuity, chief complaint, patient visit frequency, and ED volume. Because payor is not generally known at the time of triage, we interpreted it as a proxy for other observable characteristics that may influence bed assignment. We estimated a Cox proportional hazards model of hallway bed assignment on length of stay.

**Results:**

Median patient age was 53. 54.0% of visits were by women. 42.1% of visits were paid primarily by private payors, 37.1% by Medicare, and 20.7% by Medicaid. A total of 16.2% of visits were assigned to hallway beds. Hallway bed assignment was more likely for frequent ED visitors, for lower acuity presentations, and for psychiatric, substance use, and musculoskeletal chief complaints, which were more common among visits paid primarily by Medicaid. In a logistic model controlling for these factors, as well as for other patient demographics and for the volume of recent ED arrivals, Medicaid status was nevertheless associated with 22% greater odds of assignment to a hallway bed (odds ratio 1.22, [95% confidence interval, CI, 1.18–1.26]), compared to private insurance. Visits assigned to hallway beds had longer lengths of stay than roomed visits of comparable acuity (hazard ratio for departure 0.91 [95% CI, 0.90–0.92]).

**Conclusion:**

We find evidence of social determinants of hallway bed use, likely involving epidemiologic, clinical, and operational factors. Even after accounting for different distributions of chief complaints and for more frequent ED use by the Medicaid population, as well as for other visit characteristics known at the time of triage, visits paid primarily by Medicaid retain a disproportionate association with hallway bed assignment. Further research is needed to eliminate potential bias in the use of hallway beds. [West J Emerg Med. 2020;21(4)949–958.]

## INTRODUCTION

When emergency department (ED) patient volume exceeds room capacity, patients may be seen in hallway beds rather than in dedicated examination rooms. Hallway bed use increases with overall hospital crowding.^1^ Because hallway beds lack privacy compared to dedicated rooms, patients assigned to hallway beds may receive inadequate history-taking, particularly on sensitive subjects, or may be less willing to disclose information to providers. Providers may be less inclined to perform a thorough physical examination in the hallway, and patients may be less comfortable with any examination performed. In a recent survey, emergency physicians reported diagnostic errors and delays associated with hallway beds, with particular deficits for the diagnosis of self-harm, domestic violence, human trafficking, and substance abuse.^2^

Treating sick patients in hallway beds has been identified as a risk for preventable adverse outcomes, and disciplinary and legal action against providers.^3^ Even hand hygiene among ED staff has been found to be poorer in hallway care areas than in dedicated rooms.^4^ Placement in an ED hallway bed is associated with lower patient satisfaction, lower likelihood of recommending the ED to others, and a poorer assessment of a patient’s overall hospital experience.^5^,^6^ Because satisfied patients are more likely to comply with medical advice, to return for recommended follow-up, and to communicate effectively with their physicians,^7^,^8^ patients seen in hallway beds may also be at risk of poorer downstream outcomes.

Because of the provisional nature of hallway beds, hospitals may lack objective policies guiding the assignment of patients to hallway beds, risking bias in the selection of patients for these less desirable and clinically inferior beds. Clinical appropriateness dictates that, if hallway beds are to be used, patients should be assigned to these beds solely on the basis of complaints amenable to adequate care in the hall rather than in a dedicated room.^9^ In reality, factors other than clinical appropriateness affect patient trajectories throughout the healthcare system, with racial and ethnic minorities and the poor less likely to receive appropriate care in a number of venues.^10^ Patients’ insurance status (eg, private, Medicare, Medicaid, or uninsured) both directly affects the services and dispositions available to patients, and additionally serves as a proxy for socioeconomic status (ie, patients on Medicaid are more likely to be poor than patients with private insurance). Insurance status has been identified as a significant predictor of outcomes ranging from stroke treatment and recovery,^11^ to the length of ED boarding for patients requiring psychiatric hospitalization, with Medicaid patients having significantly longer ED stays than privately insured patients.^12^

Our objective was to determine whether socioeconomic factors influence which visits are assigned to hallway beds, independent of patients’ clinical characteristics at triage. In particular, we investigate whether a visit’s primary payor (as a marker of patient socioeconomic status) and patient race affect the likelihood of being triaged to a hallway bed rather than to a dedicated room, and the extent to which any such disparities may be attributed to clinical or nonclinical characteristics of visits at the time of triage. Our secondary aim was to characterize the effect of hallway bed assignment on length of stay (LOS).

## METHODS

### Study Design, Setting, and Participants

We performed a retrospective study of all visits to the adult acute care areas of two large, academic EDs from January 1, 2013–December 31, 2016. The adult acute care area of ED A had 52 roomed beds and a flexible number of hallway spaces. The adult acute care area of ED B consisted of 23 dedicated beds in private or semiprivate rooms, and five hallway spaces. In both EDs, visits were assigned to hallway beds when roomed beds were not available. The majority of patients assigned to hallway beds were seen in the hallway bed for the duration of their visit. At both sites, hallway beds were used as final sites of patient workup and management, rather than as areas for patients to wait for roomed beds.

**Population Health Research Capsule**

What do we already know about this issue?

*Hallway beds in the emergency department (ED) are associated with lower patient satisfaction and inferior care*.

What was the research question?

What determines which patients are placed in ED hallway beds, rather than in dedicated exam rooms?

What was the major finding of the study?

*Medicaid patients are more likely than comparable privately insured patients to be placed in hallway beds*.

How does this improve population health?

*Hallway beds disproportionately burden the poor through multiple mechanisms. Further research can help reduce any inappropriate bias in bed assignment*.

We included all visits to the adult acute areas of the two EDs from January 1, 2013–December 31, 2016, for which basic demographic data (age, gender, race), chief complaint, and primary payor (Medicaid, Medicare, or private insurance) were recorded. We excluded 12.2% of all visits due to absence of an unambiguous primary payor (including no insurance).

### Measurements and Outcomes

For each visit, we observed patient age, gender, race, ethnicity, and insurance status (ie, Medicaid, Medicare, or private insurance), as well as time and date of arrival, illness acuity level at triage (1–5), chief complaint, and final diagnosis by *International Classification of Disease*, 9^th^ revision, (ICD-9) code and category. For each visit, we calculated the number of same-ED arrivals in the preceding three hours, as a dynamic measure of ED volume. We also calculated the number of preceding visits by the same patient during the study period (2013–2016). The primary outcome was the bed type to which each visit was first triaged (roomed or hallway). Because only a small proportion (under 4%) of visits moved from a hallway bed to a roomed bed or vice versa during a visit, we used first assigned bed as our primary outcome. Our secondary outcome was LOS in the ED (arrival to departure, with auxiliary analyses for bed assignment to disposition decision).

### Statistical Analysis

We calculated the proportion of acute care visits of a given triage acuity level (1–5, where 1 is most urgent and 5 is least urgent) initially triaged to a hallway bed. We stratified this analysis by primary payor (Medicaid, Medicare, or private insurance), and assessed for differences in proportions of visits assigned to hallway beds by payor, using two-sample tests for equality of proportions. We performed analogous analyses stratifying by chief complaint, and by patient-stated primary race (Asian, Black, White, or other). We calculated confidence intervals (CI) for the ratios of binomial parameters (such as rates of assignment to hallway beds) using a skewness-corrected likelihood score-based method.^13^ We calculated Pearson’s correlation coefficients between visit number for a given patient (ie, the number of visits including the present visit for a given patient, 2013–2016), and the probability of hallway bed assignment, separately for each payor category. As described below, we controlled for a wide range of factors that might confound these associations.

In a logistic model of ED visits, we regressed hallway bed assignment on patient- and visit-level factors including the following: patient age; gender; race; ethnicity; and payor (indicator variables for Medicare, Medicaid, or private insurance); triage acuity (in reverse ordinal specification, such that a higher value in the model reflects greater clinical acuity); chief complaint (indicator variables for the 40 most common complaints, and an ‘Other’ category encompassing all other complaints); the number of same-ED arrivals in the three hours preceding a given visit (as a measure of momentary ED volume); and the number of visits to date from the patient associated with a given visit (as a dynamic, patient-level measure of ED use). We estimated robust standard errors, clustered by patient.

We calculated median LOS (time from ED arrival to departure) in the ED by triage acuity and bed type (roomed vs hallway), and used the Wilcoxon rank-sum test to assess for differences in median LOS. We estimated a Cox proportional hazards model for the effect of hallway bed assignment on LOS, controlling for triage acuity, age, gender, race, Hispanic ethnicity, ED volume, and chief complaint. In auxiliary models, we assessed the robustness of this result to different specifications of LOS (arrival to departure vs bed assignment to disposition decision).

In auxiliary analyses, we assessed for differences in hallway bed assignment by diagnosis, coded by ICD-9 diagnostic category. Because these diagnoses are assigned after triage decisions are made, we did not include diagnosis in our primary analyses of hallway bed assignment, as this would entail conditioning on post-triage variables, and thus induce post-exposure bias.^14^

All analyses were performed in *R*, version 3.6 (R Project for Statistical Computing). The study was approved by the institutional review boards of Stanford Health Care and Beth Israel Deaconess Medical Center.

## RESULTS

### Subject Characteristics

We observed 332,919 adult visits, across 189,326 patients, to our two EDs from January 1, 2013–December 31, 2016. Of these visits, we studied the 292,170 encounters for which a clear primary payor was identified in one of three categories (private, Medicaid, or Medicare), and for which relevant visit characteristics (triage acuity, chief complaint, time and date of visit) and patient demographics (gender, age, race, ethnicity) were recorded. The median age of patients at time of visit was 53 years, and 54.0% of visits were by female patients. Of the total visits, 42.1% had a private primary payor, 37.1% were primarily paid by Medicare, and 20.7% were primarily paid by Medicaid. With regard to race, 57.3% of patients identified as White, 18.1% as Black, 7.2% as Asian, and 17.4% identified as a different race or did not identify a race.

### Main Results

**Figure 1:**
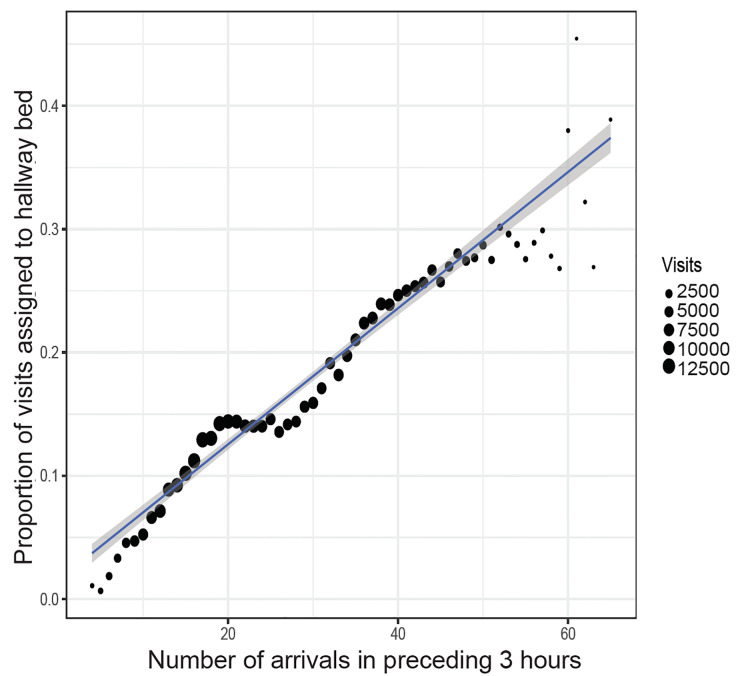
Hallway bed use and recent ED arrivals. For each visit, we calculated the number of same-ED patient arrivals in the preceding three hours. The likelihood of hallway bed assignment increases linearly with the number of recent ED arrivals, which reflects overall demand for beds. The blue line gives a visit-weighted line of best fit, and the shaded gray band shows the 95% confidence band around this estimate. On average, an additional 10 arrivals in the preceding 3 hours is associated with a 5.5% increase (95% confidence interval, 5.2% – 5.8%) in the probability of a new arrival being assigned to a hallway bed.

The proportion of adult acute care visits assigned to hallway beds was 16.2% overall, ranging from a mean of 4.2% on Mondays between 5–6 am, to 25.6% on Mondays between 3–4 pm (Figure S1). The proportion of visits assigned to hallway beds was strongly correlated with the number of same-ED arrivals in the preceding three hours, with an additional 10 arrivals in the preceding three hours associated with a 5.5% increase in the probability of a new arrival being assigned to a hallway bed (Figure 1). Acuity level 1–2 (higher acuity) visits were more likely to be triaged to roomed beds, and level 3–5 (lower acuity) visits were more likely to be triaged to hallway beds (Figure S2). There were very few level 5 visits in these data because level 5 visits were not generally seen in the acute care areas of the study EDs, but were instead triaged to separate “fast track” areas.

**Figure 2:**
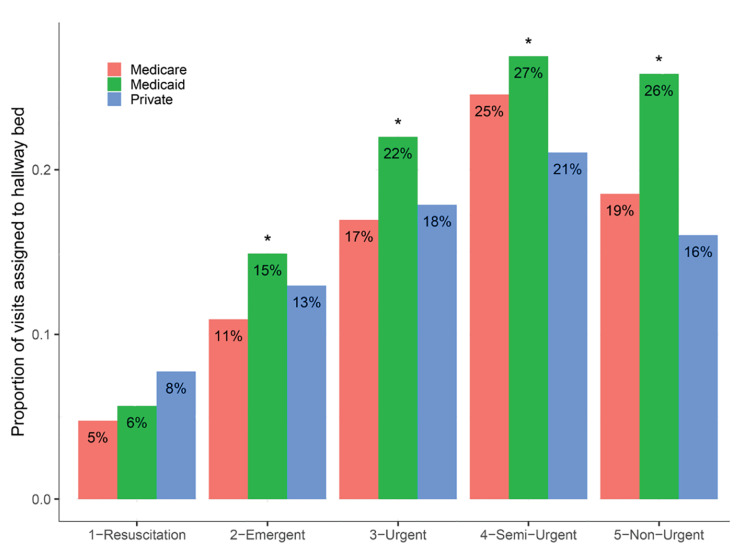
At triage acuity levels 2–5 (95.1% of visits), visits paid primarily by Medicaid were more likely to be assigned to hallway beds, compared to visits paid by Medicare or private insurance. Asterisks denote significant differences in proportions at p<0.01, comparing Medicaid visits to pooled Medicare and privately insured visits. The small proportion of Emergency Severity Index 1 visits assigned to hallway beds were predominantly stroke code activations, which are assigned to hallway beds in anticipation of imminent transportation to radiology.

At triage acuity levels 2–5 (95.1% of visits), visits paid primarily by Medicaid were more likely to be assigned to hallway beds, compared to visits paid by Medicare or private insurance (Figure 2). For instance, at triage acuity level 3 (58.5% of visits), Medicaid visits were 25.9% more likely (95% CI, 23.2% – 28.8%) to be assigned to hallway beds, compared to pooled Medicare and privately insured visits. The pattern was similar at both sites (Figure S3). We interpreted Medicaid status as a proxy for patient socioeconomic status, rather than as a direct causal factor itself, since payor is not generally known at the time of bed assignment, but is highly correlated with socioeconomic status and its potentially observable markers.

In analogous unadjusted comparisons, visits of Black patients were more likely than visits by patients of other races to be assigned to hallway beds at triage acuity levels 2–4: at level 3, Black patients were 20.7% more likely (95% CI, 18.0% – 23.5%) than patients of other races to be assigned a hallway bed, although the effect of race was more variable than the effect of insurer across our sites (Figure S4). Although Black patients were more likely than Asian or White patients to be insured by Medicaid (Figure S5), the relationship between race, insurance status, and hallway bed assignment was complex (Figure S6), and Black race was not an independently significant predictor of hallway bed use after accounting for all observable visit characteristics (Table 1), as detailed below.

**Figure 3:**
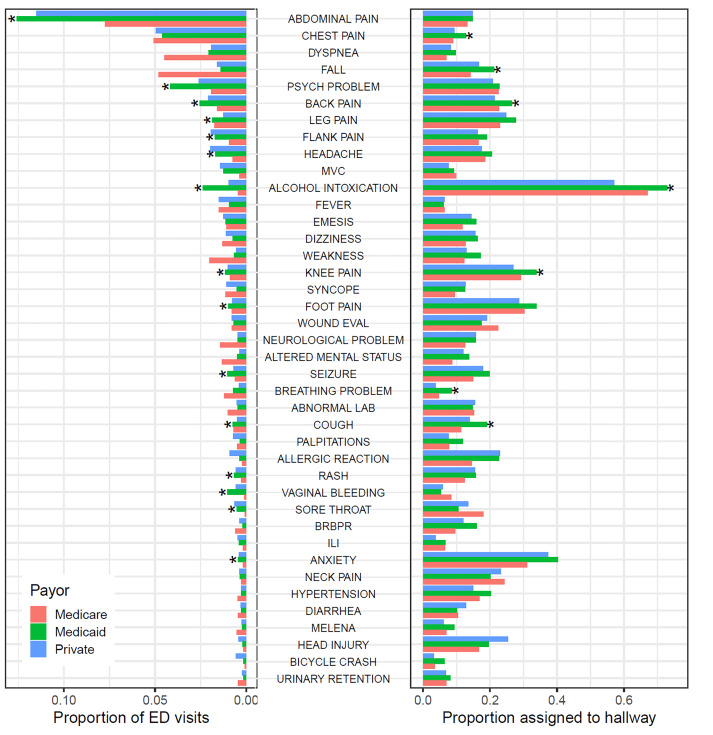
Rates of emergency department visits and hallway bed assignment, by payor and chief complaint. The 40 commonest chief complaints are shown, which collectively account for half of all visits. The left panel shows the proportion of ED visits accounted for by each complaint, stratified by payor. The right panel shows the proportion of visits of a given chief complaint assigned a hallway bed, again stratified by payor. Asterisks denote differences in proportions significant at p < 0.001 (to account for multiple comparisons), comparing Medicaid patients to Medicare and privately insured patients, pooled. Medicaid patients are more likely than patients insured by Medicare or private insurance to present with certain complaints including abdominal pain, psychiatric problems or anxiety, headache, alcohol intoxication, and back, leg, flank, or knee pain (left panel). For some of these complaints, such as psychiatric problems, Medicaid patients are not more likely to be assigned to hallway beds, conditional on complaint. For others, such as alcohol intoxication, Medicaid patients are more likely to be assigned to hallway beds, even accounting for higher prevalence of the complaint (right panel). *MVC*, motor vehicle collision; *ILI*, influenza like illness; *BRBPR*, bright red blood per rectum.

Because chief complaints may be differentially amenable to evaluation in hallway beds, and because different populations have different distributions of chief complaints, chief complaint may mediate the bivariate relationship between insurance status and hallway bed assignment we describe above. Figure 3 shows the proportion of ED visits accounted for by each of the 40 most common chief complaints, as well as the proportion of visits with a given chief complaint assigned to hallway beds, with both sets of analyses stratified by primary visit payor (Medicare, Medicaid, or private insurance). Medicaid patients were more likely than patients insured by Medicare or private insurance to present with certain complaints including abdominal pain, psychiatric problems or anxiety, headache, alcohol intoxication, and back, leg, flank, or knee pain. For some of these complaints, such as psychiatric problems, Medicaid patients were not more likely to be assigned to hallway beds, conditional on complaint. For these complaints, higher rates of assignment of Medicaid patients to hallway beds may be explained by their presenting at higher rates with hallway-amenable complaints. For other complaints, such as alcohol intoxication, Medicaid patients were more likely to be assigned to hallway beds, even accounting for higher prevalence of the complaint.

**Figure 4:**
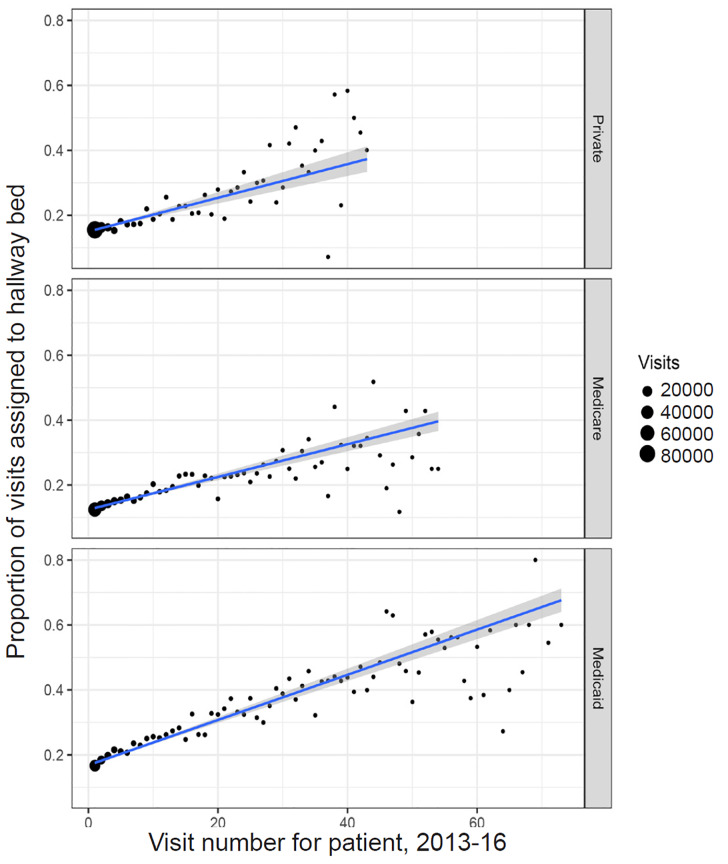
Proportion of visits assigned to hallway bed, by emergency department (ED) visit number for a given patient, 2013–2016. More frequent ED users are more likely to be assigned to hallway beds. Plots depict the proportions of visits of a given “visit number” (the number of visits up to and including the present visit by the patient associated with the present visit, in the study period, 2013–2016) assigned to hallway beds. Medicaid patients are likelier than patients with Medicare or private insurance to be frequent users of the ED (eg, with many more patients with 40 or more visits during the study period, bottom panel). Still, the correlation between visit number and hallway bed assignment is stronger for Medicaid patients (r = 0.83) than for patients with Medicare (r = 0.67) or private insurance (r = 0.75). Blue lines denote visit-weighted lines of best fit. Point size is proportional to the number of visits of a given type.

Because differently insured populations have differential access to non-ED options for care, frequency of ED use may also confound the relationship between insurance status and hallway bed assignment. If Medicaid patients are more frequent users of ED services, for instance, and if “frequent fliers” of any insurance status are more likely to be placed in hallway beds, then a higher number of frequent ED users may account for some of the apparent relationship between Medicaid status and hallway bed assignment. Figure 4 shows the relationship between hallway bed assignment and “visit number,” ie, the number of visits preceding and including the visit of interest from the same patient, in our study period. More frequent ED users were indeed more likely to be assigned to hallway beds. Medicaid patients were likelier than patients with Medicare or private insurance to be frequent users of the ED (bottom panel). Notably, however, the correlation between visit number and hallway bed assignment was stronger for visits paid by Medicaid (*r* = 0.83) than for those paid by Medicare (*r* = 0.67) or private insurance (*r* = 0.75). Compared to first or second visits from a given patient, third or later visits from the same patient were likelier to be from older patients, from patients with Medicaid or Medicare, and for chief complaints including abdominal pain, chest pain, dyspnea, psychiatric problems, alcohol intoxication, and altered mental status (Table S1). We accounted for all of these features in our models of hallway bed assignment.

Table 1 presents a logistic model of ED visits, regressing hallway bed assignment on patient- and visit-level factors including age, gender, race, Hispanic ethnicity, payor, triage acuity, chief complaint, number of same-ED arrivals in the three hours preceding a given visit (as a measure of momentary ED volume), and the number of visits to date from the patient in question. We estimated robust standard errors, clustered by patient. Controlling for these factors, we found that visits paid primarily by Medicaid had 22% greater odds of being assigned to a hallway bed (odds ratio [OR] 1.22 [95% CI, 1.18–1.26]), compared to visits paid by private insurers. In this fully specified model, Black race was not independently predictive of hallway bed assignment (OR 1.01 [95% CI, 0.98–1.05]), compared to visits of White patients. In this model, an additional patient arrival in the preceding three hours was associated with 6% greater odds of hallway bed assignment (OR 1.06 [95% CI, 1.06 – 1.06]), and an additional prior visit from the same patient predicted 3% greater odds of hallway assignment (OR 1.03 [95% CI, 1.02–1.03]). Chief complaints associated with increased odds of hallway bed assignment included the following: alcohol intoxication; psychiatric complaints; fall; and back, neck, knee, and leg pain.

Table S2 shows a hierarchy of models of increasing complexity, of which Model 4 is the final model described above. The attenuation of the estimated OR associated with Medicaid status with the sequential introduction of chief complaint (Model 1 to Model 2), and prior patient visits (Model 3 to Model 4) supports the interpretation above, i.e., that the aggregate association between Medicaid status and hallway bed assignment, as depicted in Figure 2, is mediated in part by differential distributions of chief complaints (Figure 3), and by more frequent ED use by the Medicaid population (Figure 4), but that even after accounting for these factors, as well as for other visit characteristics known at the time of triage, visits paid primarily by Medicaid retain a disproportionate association with hallway bed assignment.

We did not condition on final diagnosis in our primary analyses to avoid introducing post-exposure bias (unlike chief complaint, final diagnosis is not known at the time of bed assignment).^14^ Nevertheless, an auxiliary analysis stratifying by ICD-9 diagnostic category showed that visits by Medicaid patients were more likely to be seen in the hallway across diagnostic categories, with particularly marked disparities for injury and poisoning, mental illness, and musculoskeletal disease (Figure S7). This analysis also recapitulates the previously described finding of prolonged “boarding” of psychiatric patients in ED hallway beds,^12^,^15^ with patients presenting with psychiatric diagnoses more likely than any other diagnostic category to be assigned to hallway beds.

### Length of Stay

**Figure 5:**
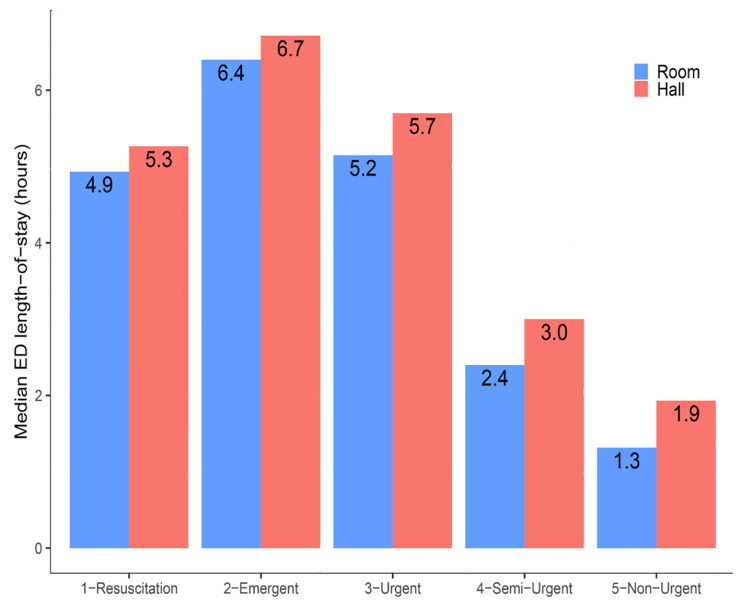
Patients assigned to hallway beds have significantly longer lengths of stay than roomed patients of the same acuity level. All differences are significant at p < 0.01 by Wilcoxon rank-sum test.

Visits assigned to hallway beds had significantly longer LOS than roomed visits of the same acuity level (Figure 5, Figure S8). In a visit-level Cox proportional hazards model of visit duration, controlling for age, gender, race, triage acuity, volume of recent arrivals, and chief complaint (Table 2), visits assigned to hallway beds had significantly longer LOS than comparable roomed visits, with a hazard ratio for ED departure of 0.91 (95% CI, 0.90–0.92). Complaints associated with significantly prolonged LOS included alcohol intoxication, psychiatric complaints, abdominal pain, and chest pain. In auxiliary models, in which we compared arrival-to-departure time and bed-assignment-to-disposition time as outcomes in otherwise identical Cox proportional hazards models estimated on the subset of patients with available times of first bed assignment and disposition decision, hazard ratios associated with hallway bed assignment were very similar with either outcome (Table S3).

## DISCUSSION

Our findings provide evidence for socioeconomic determinants of hallway bed use at two large, academic EDs. The magnitude of the association is considerable, with visits paid by Medicaid having 22% greater odds of being assigned to a hallway bed, compared to otherwise comparable visits paid by private insurance. Although Black patients were more likely than patients of other races to be assigned to hallway beds, race was not a significant predictor of hallway bed assignment after controlling for other features of visits observable at triage.

Both policy^16^ and legal precedent^17^,^18^ dictate that insurance or socioeconomic status should not affect ED triage, and that clinical personnel should not in general know the patient’s insurance status throughout initial screening and stabilization. Because evaluation in a hallway bed is associated with poorer patient satisfaction as well as with potentially inferior care,^3^–^6^ any bias in hallway bed assignment risks compounding the known disadvantages faced by the poor and by racial minorities throughout the healthcare system.

The association between Medicaid status and hallway bed assignment is likely enacted via mechanisms at different levels of analysis, of which bias in bed assignment decisions may be only one. Medicaid status is not generally known at the time of triage, and so is unlikely to directly dictate bed assignment. In our analyses, a substantial portion of the aggregate association was accounted for by higher burdens of psychiatric and substance use presentations among the Medicaid population, which likely reflect consequences of poverty, and by frequent ED users, which may reflect poorer access to primary care and specialty services among Medicaid patients. Although upstream issues of poverty and access to care cannot be solved by changes to bed assignment policies, our analysis suggests that, in many cases, a patient’s being assigned to a hallway bed can be a proxy for unmet social needs, and patients in hallway beds may be particularly likely to benefit from social work and case management services. Notably, the association between Medicaid status and hallway bed assignment persisted even after controlling for features such as chief complaint and visit frequency, suggesting other mechanisms not directly observed in our data.

We propose three avenues for further research. First, because a patient’s insurance status is not generally known before triage decisions are made, Medicaid status per se is unlikely to affect hallway bed assignment, and a qualitative study of providers making triage decisions can help identify the visit characteristics associated with insurance status that may affect bed assignment, beyond chief complaint, triage acuity level, and demographics.

Second, we propose estimating the predilection of individual triage providers to assign Medicaid patients to hallway beds. Although adequately controlling for variation in patient characteristics would require selection of only those providers triaging large numbers of visits, finding consistent and longitudinal differences among triage providers in the likelihood of assigning Medicaid patients to hallway beds would support an element of discretionary bias at the level of triage, rather than the effect of clinically relevant but unmeasured features associated with insurance status, which over large numbers of visits would be expected to be distributed similarly across the patients triaged by different providers.

Finally, to whatever extent the association between insurance status and bed assignment is driven by non-clinical bias, corrective interventions are needed. The simplest way to mitigate the inferior care and patient experience associated with hallway beds is to reduce the need for these temporary beds altogether, via hospital-wide strategies to improve the efficiency of admissions, discharges, and transfers.^19^ Since this analysis was performed, one of our study sites has moved to a new ED with only individual patient rooms and no hallway beds. Clearly, however, new facilities are not a general remedy for the use of hallway beds. To improve equity in the use of hallway beds that cannot be eliminated, triage personnel could be encouraged to consider only clinical characteristics in making bed assignments, or required to give brief justification for a decision to assign a patient to a hallway bed. A more targeted intervention would quantify the degree of possible bias in bed assignment decisions for all triage personnel, and provide each triage provider with a “scorecard” illustrating his or her historical propensity to assign Medicaid patients to hallway beds, compared to the mean propensity for triage personnel to do the same. Such an approach has been shown to reduce opioid prescribing among ED providers.^20^ After a predetermined period (e.g., one year or more), the present analyses could be repeated, including at the provider level, to assess for reductions in socioeconomic disparities, and to design more effective bias-reduction interventions in turn.

## LIMITATIONS

Our study has limitations. Although the sample size is large, the study was conducted at two centers, both large teaching hospitals, and our findings regarding hallway bed use may not generalize to other sites. Although we control for patient demographics, acuity level at triage, chief complaint, frequent visitors, and ED volume, it is conceivable that insurance status could correlate with other unrecorded but clinically relevant characteristics on which triage decisions are made. We did not observe rates of ED “boarding,” which is likely a major driver of the overall use of hallway beds. More generally, although we identify a robust association between Medicaid status and assignment to hallway beds, we do not identify all mechanisms whereby insurance status affects triage decisions. Part but not all of the association is explained by acuity, demographics, chief complaints, and frequent ED users. In general, payor is not known until patients are registered, typically after triage decisions are made. Thus, Medicaid status itself is unlikely to bias triage decisions, but rather to be correlated with some set of observable patient characteristics that affect these decisions.

## CONCLUSION

The determinants of hallway bed use are complex. Patients insured by Medicaid, a proxy for low socioeconomic status, are more likely to be assigned to hallway beds for multiple reasons. These mechanisms require further investigation to reduce the possibility of inappropriate bias in the use of hallway beds.

## Supplementary Information



## Figures and Tables

**Table 1:** Logistic regression model of hallway bed assignment.

	OR (95% CI)
Intercept	0.19 (0.18 – 0.21)
Age	1.00 (0.99 – 1.00)
Male	1.10 (1.08 – 1.13)
Triage acuity	0.60 (0.59 – 0.61)
Medicare	1.04 (1.00 – 1.07)
Medicaid	1.22 (1.18 – 1.26)
Asian	0.93 (0.89 – 0.98)
Black	1.01 (0.98 – 1.05)
Other race	1.06 (1.01 – 1.11)
Hispanic	0.92 (0.87 – 0.97)
Site B	1.51 (1.46 – 1.57)
3h arrivals	1.06 (1.06 – 1.06)
Patient visit	1.03 (1.02 – 1.03)
CC: Abdominal pain	0.87 (0.84 – 0.91)
CC: Chest pain	0.66 (0.62 – 0.70)
CC: Dyspnea	0.55 (0.51 – 0.60)
CC: Fall	1.35 (1.26 – 1.44)
CC: Psych problem	1.63 (1.50 – 1.76)
CC: Back pain	1.38 (1.29 – 1.48)
CC: Leg pain	1.57 (1.46 – 1.69)
CC: Flank pain	1.04 (0.96 – 1.13)
CC: Headache	1.15 (1.06 – 1.25)
CC: MVC	1.13 (0.98 – 1.31)
CC: Alcohol intoxication	8.50 (7.76 – 9.30)
CC: Fever	0.43 (0.38 – 0.49)
CC: Emesis	0.84 (0.76 – 0.93)
CC: Dizziness	1.09 (0.98 – 1.21)
CC: Weakness	0.91 (0.82 – 1.01)
CC: Knee pain	1.70 (1.55 – 1.86)
CC: Syncope	0.85 (0.76 – 0.96)
CC: Foot pain	1.80 (1.63 – 1.99)
CC: Wound evaluation	0.88 (0.79 – 0.98)
CC: Neurologic problem	1.56 (1.37 – 1.78)
CC: AMS	0.79 (0.69 – 0.92)
CC: Seizure	1.24 (1.10 – 1.40)
CC: Breathing problem	0.47 (0.39 – 0.57)
CC: Abnormal lab	0.96 (0.84 – 1.09)
CC: Cough	0.80 (0.70 – 0.92)
CC: Palpitations	0.59 (0.49 – 0.71)
CC: Allergic reaction	2.05 (1.81 – 2.34)
CC: Rash	0.64 (0.54 – 0.75)
CC: Vaginal bleeding	0.34 (0.27 – 0.42)
CC: Sore throat	0.60 (0.50 – 0.71)
CC: BRBPR	0.71 (0.59 – 0.86)
CC: Flu-like illness	0.21 (0.16 – 0.28)
CC: Anxiety	3.12 (2.70 – 3.59)
CC: Neck pain	1.54 (1.31 – 1.81)
CC: Hypertension	1.10 (0.93 – 1.30)
CC: Diarrhea	0.65 (0.53 – 0.79)
CC: Melena	0.52 (0.41 – 0.67)
CC: Head injury	1.50 (1.26 – 1.80)
CC: Bicycle accident	0.59 (0.41 – 0.86)
CC: Urinary retention	0.43 (0.33 – 0.56)
N	281,183
AIC	228,397

Standard errors clustered by patient. Entries denote odds ratios (OR) and 95% confidence intervals (CI).

*CC*, chief complaint; *MVC*, motor vehicle collision; *AMS*, altered medical status; *BRBPR*, bright red blood per rectum; *AIC*, Akaike information criterion.

**Table 2:** Cox proportional hazards model for time to emergency department departure (admission or discharge).

	Hazard ratio (95% CI)
Hallway bed	0.91 (0.90 – 0.92)
Triage acuity	0.74 (0.74 – 0.75)
Age	0.99 (0.99 – 0.99)
Male	1.01 (1.01 – 1.02)
Asian	1.03 (1.02 – 1.05)
Black	0.94 (0.93 – 0.95)
Other race	1.01 (0.99 – 1.02)
Hispanic	0.96 (0.94 – 0.98)
ED B	1.04 (1.03 – 1.06)
3h arrivals	0.99 (0.99 – 0.99)
CC: Abdominal pain	0.77 (0.76 – 0.78)
CC: Chest pain	0.79 (0.78 – 0.81)
CC: Dyspnea	0.96 (0.94 – 0.99)
CC: Fall	0.97 (0.94 – 0.99)
CC: Psych problem	0.36 (0.36 – 0.37)
CC: Back pain	1.02 (0.99 – 1.05)
CC: Leg pain	0.92 (0.90 – 0.95)
CC: Flank pain	0.86 (0.83 – 0.88)
CC: Headache	1.05 (1.01 – 1.08)
CC: MVC	1.61 (1.52 – 1.69)
CC: Alcohol intoxication	0.63 (0.60 – 0.65)
CC: Fever	0.86 (0.83 – 0.88)
CC: Emesis	0.78 (0.76 – 0.81)
CC: Dizziness	1.07 (1.03 – 1.11)
CC: Weakness	0.80 (0.78 – 0.83)
CC: Knee pain	1.10 (1.06 – 1.14)
CC: Syncope	1.11 (1.07 – 1.16)
CC: Foot pain	1.21 (1.16 – 1.26)
CC: Wound evaluation	1.09 (1.04 – 1.13)
CC: Neurologic problem	1.01 (0.97 – 1.05)
CC: AMS	0.87 (0.84 – 0.91)
CC: Seizure	0.93 (0.89 – 0.97)
CC: Breathing problem	0.80 (0.77 – 0.84)
CC: Abnormal lab	0.91 (0.87 – 0.95)
CC: Cough	1.20 (1.15 – 1.26)
CC: Palpitations	1.19 (1.14 – 1.25)
CC: Allergic reaction	1.84 (1.75 – 1.94)
CC: Rash	2.04 (1.94 – 2.16)
CC: Vaginal bleeding	1.15 (1.09 – 1.21)
CC: Sore throat	1.35 (1.27 – 1.43)
CC: BRBPR	1.24 (1.17 – 1.31)
CC: Flu-like illness	1.07 (1.01 – 1.14)
CC: Anxiety	0.78 (0.73 – 0.83)
CC: Neck pain	1.22 (1.15 – 1.30)
CC: Hypertension	1.48 (1.39 – 1.58)
CC: Diarrhea	0.97 (0.91 – 1.03)
CC: Melena	0.90 (0.84 – 0.95)
CC: Head injury	2.22 (2.07 – 2.39)
CC: Bicycle accident	1.19 (1.08 – 1.32)
CC: Urinary retention	1.39 (1.30 – 1.49)
N	281,143
R^2^	0.12

Standard errors clustered by patient. Entries denote hazard ratios and 95% confidence intervals (CI).

*ED*, emergency department; *CC*, chief complaint; *MVC*, motor vehicle collision; *AMS*, altered medical status; *BRBPR*, bright red blood per rectum.
