# Supplementary material for: Social Determinants of Hallway Bed Use
Source: West J Emerg Med. 2020 Jun 24;21(4):949–58. doi: 10.5811/westjem.2020.4.45976 (PMC7390564; doi:10.5811/westjem.2020.4.45976)
Supplement: Supplementary file 1 [file wjem-21-949-s001.docx]

**Supplementary Materials: Social Determinants of Hallway Bed Use**

**David A. Kim, MD, PhD^*^**

**Leon D. Sanchez, MD, MPH^†^**

**David Chiu, MD^†^**

**Ian P. Brown, MD^*^**

^*^Stanford University, Department of Emergency Medicine, Palo Alto, California

^†^Beth Israel Deaconess Medical Center, Department of Emergency Medicine, Boston, Massachusetts

[Figure S1. Hallway bed use by time of day and day of week. 2](#_Toc34826112)

[Figure S2. Hallway bed use by triage acuity. 3](#_Toc34826113)

[Figure S3. Hallway bed use by acuity and primary payor. 4](#_Toc34826114)

[Figure S4. Hallway bed use by acuity and race. 5](#_Toc34826115)

[Figure S5. Insurance status by race 6](#_Toc34826116)

[Figure S6. Interactions between insurance status, race, and triage acuity in hallway bed assignment. 7](#_Toc34826117)

[Figure S7. Hallway bed use by category of final diagnosis. 8](#_Toc34826118)

[Figure S8. Median length-of-stay by acuity and bed type. 9](#_Toc34826119)

[Table S1. Characteristics of visits by frequent visitors. 10](#_Toc34826120)

[Table S2. Logistic regression models for hallway bed assignment. 11](#_Toc34826121)

[Table S3. Cox proportional hazards regression models comparing arrival-to-departure and bed-assignment-to-disposition times as outcomes. 12](#_Toc34826122)

Figure S1. Hallway bed use by time of day and day of week. Across both sites, the percentage of adult acute care area visits roomed in hallway beds was 16.2% overall, ranging from an average of 4.2% on Mondays between 5-6am, to 25.6% on Mondays between 3-4pm (upper panel). The pattern is similar at both sites, with a higher overall proportion of visits assigned to hallway beds at ED A (lower panel).

Figure S2. Hallway bed use by triage acuity. Acuity level 1-2 (higher acuity) visits were more likely to be triaged to roomed beds. Level 3-5 (lower acuity) visits were more likely to be triaged to hallway beds. All differences in proportions are significant at *p* < 0.01. The small proportion of level 1 visits assigned to hallway beds were predominantly stroke code activations, which are assigned to hallway beds in anticipation of imminent transportation to radiology. There are very few level 5 visits in these data because level 5 visits are not generally seen in the acute care areas of the study EDs, but are instead triaged to separate ‘fast track’ areas.

Figure S3. Hallway bed use by acuity and primary payor. At both sites, Medicaid visits were more likely to be assigned to hallway beds, compared to visits paid by Medicare or private insurance. Asterisks denote significant differences in proportions at *p* < 0.01, comparing Medicaid visits to pooled Medicare and privately insured visits.

Figure S4. Hallway bed use by acuity and race. At triage acuity levels 2-4 (95.1% of visits), Black patients were more likely to be assigned to hallway beds, compared to other races (upper panel). The lower panel stratifies the analysis by study site. At triage acuity level 3 (59.0% of visits), Black patients were more likely to be assigned to hallway beds at both sites. At ED B, Black patients were more likely to be assigned to hallway beds at acuity levels 2 and 4, as well. Asterisks denote significant differences in proportions at *p* < 0.01, comparing Black patients to other races, pooled.

Figure S5. Insurance status by race**.** Black patients and patients identifying as non-White and non-Asian (‘Other’, above) are more likely than Asian or White patients to be insured by Medicaid (all pairwise comparisons of proportions insured by Medicaid are significant at *p* < 0.01).

**

Figure S6. Interactions between insurance status, race, and triage acuity in hallway bed assignment. In logistic regression models controlling for age, sex, race, ED volume, triage acuity, and chief complaint, Medicaid status was associated with 22% greater odds of assignment to a hallway bed (OR 1.22 [95% CI, 1.18-1.26]), compared to privately insured patients. Black race was not independently associated with hallway bed assignment (OR 1.01 [95% CI, 0.98 - 1.05]). The upper panel presents pooled results for hallway bed assignment by payor, race, and acuity; the lower panel stratifies the same analysis by study site.

Figure S7. Hallway bed use by category of final diagnosis. Medicaid visits are more likely to be seen in the hallway across most ICD-9 diagnostic categories. Asterisks denote significant differences in proportions at p < 0.001, comparing Medicaid visits to pooled Medicare and privately insured visits.

Figure S8. Median length-of-stay by acuity and bed type. At both sites, visits assigned to hallway beds had significantly longer lengths-of-stay than roomed visits of the same acuity level. All pairwise differences are significant at *p* < 0.01 by Wilcoxon rank sum tests.

Table S1. Characteristics of visits by frequent visitors. 28% of visits are from patients with two or more prior visits in the study period. Values give proportions for each variable, with the exception of number of visits (count), and age and acuity (means). Asterisks denote differences statistically significant at *p* < 0.001.

|  | **1st or 2nd visit** | **3rd+ visit** | **p < 0.001** |
| --- | --- | --- | --- |
| Number of visits | 244,903 | 88,016 |  |
| Hallway | 0.151 | 0.191 | * |
| Age (mean) | 51.071 | 56.371 | * |
| Male | 0.462 | 0.456 |  |
| Acuity (mean) | 2.692 | 2.662 | * |
| Medicaid | 0.157 | 0.252 | * |
| Medicare | 0.285 | 0.440 | * |
| Private | 0.417 | 0.239 | * |
| CC: Abdominal pain | 0.097 | 0.106 | * |
| CC: Chest pain | 0.046 | 0.052 | * |
| CC: Dyspnea | 0.024 | 0.037 | * |
| CC: Fall | 0.029 | 0.022 | * |
| CC: Psych problem | 0.024 | 0.031 | * |
| CC: Back pain | 0.020 | 0.020 |  |
| CC: Leg pain | 0.014 | 0.019 | * |
| CC: Flank pain | 0.015 | 0.016 |  |
| CC: Headache | 0.015 | 0.013 |  |
| CC: MVC | 0.018 | 0.002 | * |
| CC: Alcohol intoxication | 0.010 | 0.021 | * |
| CC: Fever | 0.012 | 0.014 | * |
| CC: Emesis | 0.010 | 0.014 | * |
| CC: Dizziness | 0.011 | 0.008 | * |
| CC: Weakness | 0.009 | 0.014 | * |
| CC: Knee pain | 0.010 | 0.011 |  |
| CC: Syncope | 0.011 | 0.006 | * |
| CC: Foot pain | 0.007 | 0.011 | * |
| CC: Wound evaluation | 0.007 | 0.010 | * |
| CC: Neurologic problem | 0.008 | 0.006 | * |
| CC: AMS | 0.006 | 0.011 | * |
| CC: Seizure | 0.007 | 0.009 | * |
| CC: Breathing problem | 0.006 | 0.008 | * |
| CC: Abnormal lab | 0.006 | 0.008 | * |
| CC: Cough | 0.006 | 0.007 |  |
| CC: Palpitations | 0.005 | 0.005 |  |
| CC: Allergic reaction | 0.006 | 0.003 | * |
| CC: Rash | 0.005 | 0.004 | * |
| CC: Vaginal bleeding | 0.006 | 0.003 | * |
| CC: Sore throat | 0.005 | 0.003 | * |
| CC: BRBPR | 0.004 | 0.005 | * |
| CC: Flu-like illness | 0.003 | 0.004 |  |
| CC: Anxiety | 0.003 | 0.004 |  |
| CC: Neck pain | 0.004 | 0.003 |  |
| CC: Hypertension | 0.003 | 0.003 |  |
| CC: Diarrhea | 0.003 | 0.004 | * |
| CC: Melena | 0.003 | 0.003 |  |
| CC: Head injury | 0.004 | 0.001 | * |
| CC: Bicycle accident | 0.004 | 0.000 | * |
| CC: Urinary retention | 0.003 | 0.003 |  |

Table S2. Logistic regression models for hallway bed assignment. Standard errors clustered by patient. Entries denote odds ratios and 95% confidence intervals.

|  | **Model 1** | **Model 2** | **Model 3** | **Model 4** |
| --- | --- | --- | --- | --- |
| Intercept | 1.20 (1.13 - 1.28) | 1.04 (0.98 - 1.11) | 0.20 (0.19 - 0.22) | 0.19 (0.18 - 0.21) |
| Age | 0.99 (0.99 - 1.00) | 1.00 (1.00 - 1.00) | 1.00 (0.99 - 1.00) | 1.00 (0.99 - 1.00) |
| Male | 1.15 (1.12 - 1.19) | 1.09 (1.07 - 1.12) | 1.12 (1.09 - 1.15) | 1.10 (1.08 - 1.13) |
| Triage acuity | 0.63 (0.62 - 0.64) | 0.64 (0.63 - 0.65) | 0.60 (0.59 - 0.61) | 0.60 (0.59 - 0.61) |
| Medicare | 1.12 (1.07 - 1.17) | 1.10 (1.06 - 1.14) | 1.09 (1.05 - 1.13) | 1.04 (1.00 - 1.07) |
| Medicaid | 1.38 (1.32 - 1.44) | 1.29 (1.24 - 1.33) | 1.29 (1.25 - 1.34) | 1.22 (1.18 - 1.26) |
| Asian | 0.89 (0.84 - 0.93) | 0.93 (0.89 - 0.97) | 0.92 (0.87 - 0.96) | 0.93 (0.89 - 0.98) |
| Black | 1.04 (0.99 - 1.09) | 1.04 (1.00 - 1.08) | 1.06 (1.02 - 1.10) | 1.01 (0.98 - 1.05) |
| Other race | 1.02 (0.97 - 1.07) | 1.05 (1.00 - 1.10) | 1.04 (0.99 - 1.09) | 1.06 (1.01 - 1.11) |
| Hispanic | 0.93 (0.87 - 0.98) | 0.94 (0.89 - 0.99) | 0.93 (0.88 - 0.99) | 0.92 (0.87 - 0.97) |
| Site B | 0.57 (0.56 - 0.59) | 0.59 (0.57 - 0.61) | 1.48 (1.42 - 1.54) | 1.51 (1.46 - 1.57) |
| 3h arrivals |  |  | 1.06 (1.06 - 1.06) | 1.06 (1.06 - 1.06) |
| Patient visit |  |  |  | 1.03 (1.02 - 1.03) |
| CC: Abdominal pain |  | 0.86 (0.82 - 0.89) | 0.89 (0.86 - 0.92) | 0.87 (0.84 - 0.91) |
| CC: Chest pain |  | 0.66 (0.62 - 0.71) | 0.67 (0.63 - 0.72) | 0.66 (0.62 - 0.70) |
| CC: Dyspnea |  | 0.54 (0.50 - 0.59) | 0.56 (0.51 - 0.60) | 0.55 (0.51 - 0.60) |
| CC: Fall |  | 1.22 (1.14 - 1.30) | 1.33 (1.24 - 1.42) | 1.35 (1.26 - 1.44) |
| CC: Psych problem |  | 1.68 (1.56 - 1.81) | 1.67 (1.55 - 1.80) | 1.63 (1.50 - 1.76) |
| CC: Back pain |  | 1.29 (1.21 - 1.38) | 1.37 (1.28 - 1.47) | 1.38 (1.29 - 1.48) |
| CC: Leg pain |  | 1.57 (1.46 - 1.68) | 1.60 (1.48 - 1.72) | 1.57 (1.46 - 1.69) |
| CC: Flank pain |  | 0.98 (0.91 - 1.07) | 1.05 (0.97 - 1.14) | 1.04 (0.96 - 1.13) |
| CC: Headache |  | 1.12 (1.04 - 1.22) | 1.15 (1.06 - 1.25) | 1.15 (1.06 - 1.25) |
| CC: MVC |  | 1.03 (0.90 - 1.19) | 1.09 (0.95 - 1.26) | 1.13 (0.98 - 1.31) |
| CC: Alcohol intoxication |  | 8.50 (7.66 - 9.43) | 9.80 (8.88 - 10.81) | 8.50 (7.76 - 9.30) |
| CC: Fever |  | 0.42 (0.37 - 0.48) | 0.43 (0.38 - 0.49) | 0.43 (0.38 - 0.49) |
| CC: Emesis |  | 0.79 (0.71 - 0.87) | 0.86 (0.78 - 0.95) | 0.84 (0.76 - 0.93) |
| CC: Dizziness |  | 1.04 (0.94 - 1.15) | 1.08 (0.97 - 1.20) | 1.09 (0.98 - 1.21) |
| CC: Weakness |  | 0.92 (0.83 - 1.02) | 0.91 (0.82 - 1.01) | 0.91 (0.82 - 1.01) |
| CC: Knee pain |  | 1.64 (1.51 - 1.78) | 1.69 (1.55 - 1.85) | 1.70 (1.55 - 1.86) |
| CC: Syncope |  | 0.81 (0.72 - 0.92) | 0.84 (0.74 - 0.94) | 0.85 (0.76 - 0.96) |
| CC: Foot pain |  | 1.75 (1.60 - 1.92) | 1.81 (1.64 - 2.00) | 1.80 (1.63 - 1.99) |
| CC: Wound evaluation |  | 0.94 (0.84 - 1.04) | 0.88 (0.79 - 0.98) | 0.88 (0.79 - 0.98) |
| CC: Neurologic problem |  | 1.47 (1.29 - 1.67) | 1.55 (1.36 - 1.76) | 1.56 (1.37 - 1.78) |
| CC: AMS |  | 0.78 (0.68 - 0.90) | 0.80 (0.69 - 0.92) | 0.79 (0.69 - 0.92) |
| CC: Seizure |  | 1.26 (1.12 - 1.41) | 1.27 (1.13 - 1.43) | 1.24 (1.10 - 1.40) |
| CC: Breathing problem |  | 0.46 (0.38 - 0.56) | 0.47 (0.39 - 0.57) | 0.47 (0.39 - 0.57) |
| CC: Abnormal lab |  | 1.07 (0.95 - 1.22) | 0.96 (0.85 - 1.09) | 0.96 (0.84 - 1.09) |
| CC: Cough |  | 0.76 (0.67 - 0.88) | 0.80 (0.70 - 0.92) | 0.80 (0.70 - 0.92) |
| CC: Palpitations |  | 0.53 (0.45 - 0.64) | 0.59 (0.49 - 0.70) | 0.59 (0.49 - 0.71) |
| CC: Allergic reaction |  | 1.84 (1.63 - 2.09) | 2.04 (1.80 - 2.32) | 2.05 (1.81 - 2.34) |
| CC: Rash |  | 0.63 (0.54 - 0.73) | 0.62 (0.53 - 0.73) | 0.64 (0.54 - 0.75) |
| CC: Vaginal bleeding |  | 0.33 (0.26 - 0.41) | 0.33 (0.26 - 0.42) | 0.34 (0.27 - 0.42) |
| CC: Sore throat |  | 0.54 (0.46 - 0.64) | 0.59 (0.49 - 0.70) | 0.60 (0.50 - 0.71) |
| CC: BRBPR |  | 0.70 (0.58 - 0.84) | 0.74 (0.61 - 0.88) | 0.71 (0.59 - 0.86) |
| CC: Flu-like illness |  | 0.20 (0.15 - 0.27) | 0.21 (0.16 - 0.28) | 0.21 (0.16 - 0.28) |
| CC: Anxiety |  | 2.77 (2.40 - 3.18) | 3.12 (2.70 - 3.60) | 3.12 (2.70 - 3.59) |
| CC: Neck pain |  | 1.36 (1.16 - 1.59) | 1.52 (1.30 - 1.79) | 1.54 (1.31 - 1.81) |
| CC: Hypertension |  | 1.14 (0.97 - 1.35) | 1.07 (0.91 - 1.26) | 1.10 (0.93 - 1.30) |
| CC: Diarrhea |  | 0.65 (0.54 - 0.80) | 0.66 (0.54 - 0.81) | 0.65 (0.53 - 0.79) |
| CC: Melena |  | 0.54 (0.42 - 0.69) | 0.52 (0.41 - 0.67) | 0.52 (0.41 - 0.67) |
| CC: Head injury |  | 1.48 (1.25 - 1.76) | 1.48 (1.23 - 1.77) | 1.50 (1.26 - 1.80) |
| CC: Bicycle accident |  | 0.58 (0.40 - 0.85) | 0.58 (0.40 - 0.84) | 0.59 (0.41 - 0.86) |
| CC: Urinary retention |  | 0.38 (0.29 - 0.50) | 0.43 (0.33 - 0.55) | 0.43 (0.33 - 0.56) |
|  |  |  |  |  |
| N | 281,194 | 281,194 | 281,183 | 281,183 |
| AIC | 245,333 | 239,488 | 229,437 | 228,397 |

Table S3. Cox proportional hazards regression models comparing arrival-to-departure and bed-assignment-to-disposition times as outcomes. Both models are restricted to the subset of visits with first bed assignment and disposition decision times recorded. Standard errors clustered by patient. Entries denote hazard ratios and 95% confidence intervals.

|  | **Arrival to departure** | **Bed assignment to disposition decision** |
| --- | --- | --- |
| Hallway bed | 0.93 (0.91 - 0.95) | 0.94 (0.92 - 0.96) |
| Triage acuity | 0.63 (0.62 - 0.64) | 0.83 (0.82 - 0.85) |
| Age | 0.99 (0.99 - 0.99) | 1.00 (1.00 - 1.00) |
| Male | 0.99 (0.98 - 1.00) | 1.05 (1.03 - 1.06) |
| Asian | 1.01 (0.99 - 1.03) | 1.03 (1.01 - 1.05) |
| Black | 0.94 (0.91 - 0.96) | 0.91 (0.89 - 0.93) |
| Other race | 0.97 (0.95 - 1.00) | 0.98 (0.96 - 1.00) |
| Hispanic | 0.98 (0.96 - 1.01) | 0.97 (0.95 - 0.99) |
| 3h arrivals | 0.98 (0.98 - 0.98) | 1.00 (1.00 - 1.00) |
| CC: Abdominal pain | 0.75 (0.74 - 0.77) | 0.68 (0.67 - 0.70) |
| CC: Chest pain | 1.02 (0.99 - 1.05) | 1.11 (1.07 - 1.14) |
| CC: Dyspnea | 0.87 (0.83 - 0.90) | 1.07 (1.03 - 1.11) |
| CC: Fall | 0.97 (0.93 - 1.01) | 0.82 (0.79 - 0.85) |
| CC: Psych problem | 0.45 (0.43 - 0.47) | 0.74 (0.71 - 0.77) |
| CC: Back pain | 1.00 (0.95 - 1.05) | 0.82 (0.78 - 0.86) |
| CC: Leg pain | 0.91 (0.87 - 0.96) | 0.86 (0.82 - 0.90) |
| CC: Flank pain | 0.89 (0.85 - 0.95) | 0.74 (0.70 - 0.78) |
| CC: Headache | 1.08 (1.02 - 1.14) | 0.88 (0.83 - 0.93) |
| CC: MVC | 1.55 (1.44 - 1.66) | 1.22 (1.13 - 1.31) |
| CC: Alcohol intoxication | 0.73 (0.68 - 0.79) | 0.54 (0.50 - 0.58) |
| CC: Fever | 0.78 (0.75 - 0.82) | 1.15 (1.10 - 1.21) |
| CC: Emesis | 0.77 (0.73 - 0.82) | 0.78 (0.74 - 0.83) |
| CC: Dizziness | 1.13 (1.07 - 1.19) | 0.93 (0.88 - 0.98) |
| CC: Weakness | 0.77 (0.73 - 0.81) | 0.86 (0.82 - 0.91) |
| CC: Knee pain | 1.07 (0.99 - 1.15) | 0.94 (0.87 - 1.02) |
| CC: Syncope | 1.21 (1.14 - 1.28) | 1.17 (1.10 - 1.24) |
| CC: Foot pain | 1.01 (0.93 - 1.11) | 1.04 (0.95 - 1.14) |
| CC: Neurologic problem | 1.08 (1.03 - 1.13) | 1.12 (1.07 - 1.17) |
| CC: AMS | 0.74 (0.67 - 0.81) | 0.85 (0.77 - 0.92) |
| CC: Seizure | 0.86 (0.80 - 0.92) | 0.73 (0.68 - 0.78) |
| CC: Breathing problem | 0.85 (0.82 - 0.89) | 1.11 (1.06 - 1.16) |
| CC: Abnormal lab | 0.84 (0.78 - 0.90) | 1.29 (1.21 - 1.39) |
| CC: Cough | 1.08 (1.00 - 1.16) | 1.53 (1.42 - 1.65) |
| CC: Palpitations | 1.28 (1.18 - 1.40) | 1.21 (1.12 - 1.32) |
| CC: Allergic reaction | 1.91 (1.76 - 2.07) | 1.38 (1.27 - 1.49) |
| CC: Rash | 1.65 (1.49 - 1.83) | 1.83 (1.65 - 2.03) |
| CC: Vaginal bleeding | 1.11 (1.03 - 1.19) | 0.93 (0.87 - 1.00) |
| CC: Sore throat | 1.89 (1.63 - 2.21) | 1.64 (1.41 - 1.92) |
| CC: Anxiety | 0.77 (0.69 - 0.85) | 0.93 (0.84 - 1.04) |
| CC: Neck pain | 1.07 (0.96 - 1.19) | 0.92 (0.82 - 1.02) |
| CC: Hypertension | 1.51 (1.37 - 1.67) | 1.24 (1.12 - 1.37) |
| CC: Diarrhea | 0.98 (0.89 - 1.09) | 0.96 (0.87 - 1.06) |
| CC: Melena | 0.85 (0.79 - 0.91) | 1.30 (1.21 - 1.39) |
| CC: Head injury | 1.89 (1.64 - 2.16) | 1.48 (1.29 - 1.70) |
| CC: Bicycle accident | 1.20 (1.08 - 1.34) | 0.94 (0.85 - 1.05) |
| CC: Urinary retention | 1.47 (1.30 - 1.66) | 1.19 (1.06 - 1.35) |
|  |  |  |
| N | 86,997 | 86,997 |
| R^2^ | 0.12 | 0.05 |
